# 4′,5-Dihy­droxy-7-meth­oxy­flavanone dihydrate

**DOI:** 10.1107/S1600536811051221

**Published:** 2011-12-07

**Authors:** Iván Brito, Jorge Bórquez, Mario Simirgiotis, Alejandro Cárdenas, Matías López-Rodríguez

**Affiliations:** a Departamento de Química, Facultad de Ciencias Básicas, Universidad de Antofagasta, Casilla 170, Antofagasta, Chile; b Departamento de Física, Facultad de Ciencias Básicas, Universidad de Antofagasta, Casilla 170, Antofagasta, Chile; c Instituto de Bio-Orgánica ’Antonio González’, Universidad de La Laguna, Astrofísico Francisco Sánchez N°2, La Laguna, Tenerife, Spain

## Abstract

The title compound, C_16_H_14_O_5_·2H_2_O [systematic name: 5-hy­droxy-2-(4-hy­droxy­phen­yl)-7-meth­oxy­chroman-4-one dihydrate], is a natural phytoalexin flavone isolated from the native chilean species *Heliotropium taltalense* and crystallizes with an organic mol­ecule and two water mol­ecules in the asymmetric unit. The 5-hy­droxy group forms a strong intra­molecular hydrogen bond with the carbonyl group, resulting in a six-membered ring. In the crystal, the components are linked by O—H⋯O hydrogen bonds, forming a three-dimensional network. The 4-hy­droxy­phenyl benzene ring is bonded equatorially to the pyrone ring, which adopts a slightly distorted sofa conformation. The title compound is the hydrated form of a previously reported structure [Shoja (1990 ▶). *Acta Cryst.* C**46**, 1969–1971]. There are only slight variations in the mol­ecular geometry between the two compounds.

## Related literature

For the first study of the title compound, see: Narasimhachari & Seshadri (1949 ▶); Atkinson & Blakeman (1982 ▶). For its biological properties, see: Plowright *et al.* (1996 ▶); Atkinson & Blakeman (1982 ▶), Saito *et al.* (2008 ▶). For its spectroscopic properties, see: Agrawal (1989 ▶); Ogawa *et al.* (2007 ▶). For the structure of the unsolvated compound, see: Shoja (1990 ▶). For similar compounds, see: Modak *et al.* (2009 ▶). For graph-set notation, see: Bernstein *et al.* (1995 ▶). For puckering parameters, see: Cremer & Pople (1975 ▶). For mol­ecular geometry calculations, see: Macrae *et al.* (2008 ▶). 
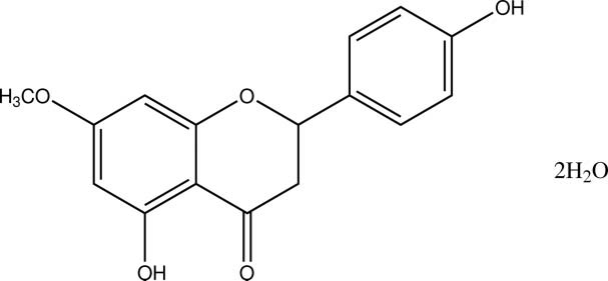


## Experimental

### 

#### Crystal data

C_16_H_14_O_5_·2H_2_O
*M* *_r_* = 322.30
Orthorhombic, 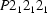

*a* = 5.0869 (10) Å
*b* = 9.4622 (19) Å
*c* = 32.318 (7) Å
*V* = 1555.6 (5) Å^3^
*Z* = 4
Mo *K*α radiation
μ = 0.11 mm^−1^
*T* = 293 K
0.20 × 0.15 × 0.03 mm

#### Data collection

Nonius KappaCCD area-detector diffractometer
9743 measured reflections
2021 independent reflections
1623 reflections with *I* > 2σ(*I*)
*R*_int_ = 0.068

#### Refinement

*R*[*F* ^2^ > 2σ(*F* ^2^)] = 0.075
*wR*(*F* ^2^) = 0.180
*S* = 1.17
2021 reflections
224 parameters
H atoms treated by a mixture of independent and constrained refinement
Δρ_max_ = 0.38 e Å^−3^
Δρ_min_ = −0.29 e Å^−3^

### 

Data collection: *COLLECT* (Nonius, 2000 ▶; cell refinement: *DENZO-SMN* (Otwinowski & Minor, 1997 ▶); data reduction: *DENZO-SMN*; program(s) used to solve structure: *SHELXS97* (Sheldrick, 2008 ▶); program(s) used to refine structure: *SHELXL97* (Sheldrick, 2008 ▶); molecular graphics: *PLATON* (Spek, 2009 ▶); software used to prepare material for publication: *WinGX* (Farrugia, 1999 ▶) and *publCIF* (Westrip, 2010 ▶).

## Supplementary Material

Crystal structure: contains datablock(s) I, global. DOI: 10.1107/S1600536811051221/kj2193sup1.cif

Structure factors: contains datablock(s) I. DOI: 10.1107/S1600536811051221/kj2193Isup2.hkl

Supplementary material file. DOI: 10.1107/S1600536811051221/kj2193Isup3.cml

Additional supplementary materials:  crystallographic information; 3D view; checkCIF report

## Figures and Tables

**Table 1:** Hydrogen-bond geometry (Å, °)

*D*—H⋯*A*	*D*—H	H⋯*A*	*D*⋯*A*	*D*—H⋯*A*
O3—H3⋯O2	0.82	1.90	2.630 (5)	147
O5—H5⋯O7	0.76 (9)	1.99 (9)	2.720 (7)	163 (7)
O6—H6*A*⋯O2^i^	0.87 (9)	2.00 (8)	2.847 (6)	166 (7)
O6—H6*B*⋯O3^ii^	0.81 (6)	2.11 (6)	2.915 (5)	174 (8)
O7—H7*A*⋯O5^i^	0.85	2.27	3.055 (7)	153
O7—H7*B*⋯O6^iii^	0.85	1.94	2.763 (6)	163

Symmetry codes: (i) 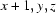; (ii) 
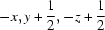
; (iii) 
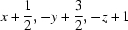
.

**Fig. 1.**
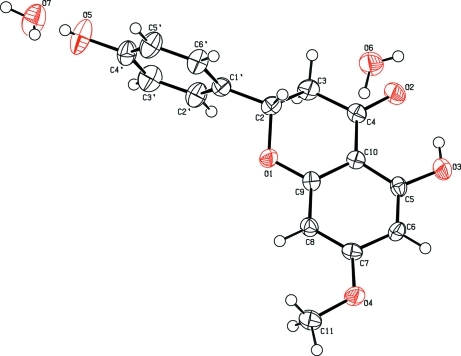
: Molecular structure of title compound. Displacement ellipsoids are shown at the 50% probability level.

**Fig. 2.**
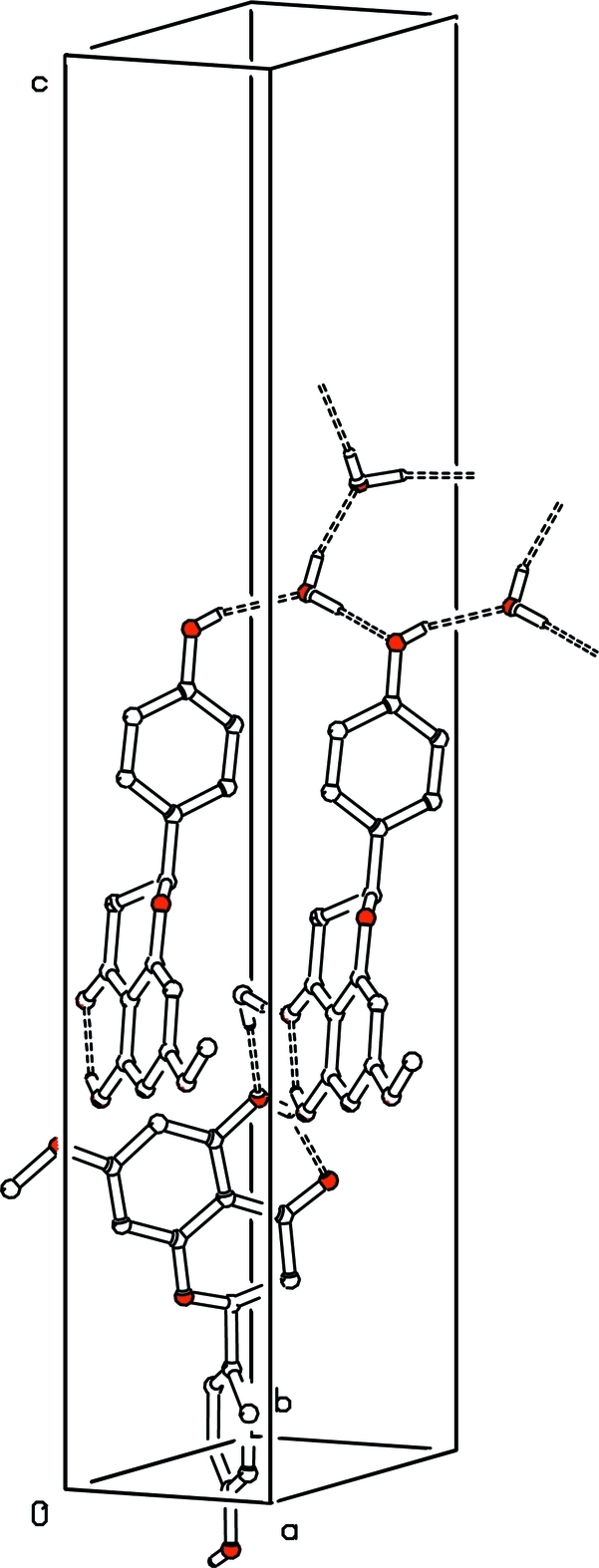
: A view of the crystal packing of the title compound, showing the intermolecular and intramolecular hydrogen bonding.

**Fig. 3.**
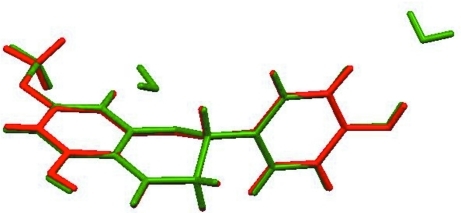
Superimposed structures for the title compound (green) and the unsolvated compound (red).

## supplementary crystallographic information

### Comment

The crystal structure of the flavanone (*S*)-sakuranetin (5,4'-di-hydroxy-7-methoxyflavanone dihydrate, C_16_H_14_O_5_.2H_2_O) which was isolated from the native Chilean shrub *Heliotropium taltalense* (*Heliotropiaceae*) collected in Quebrada de Paposo II Region of Chile, is presented in this paper. (*S*)-Sakuranetin is a methylated flavanone obtained from the bark of *Prunuspuddum* (Narasimhachari & Seshadri, 1949) and later from other *Prunus* species (Atkinson & Blakeman, 1982). This compound is a phytoalexin produced in response to infection in rice (Plowright *et al.*, 1996) with several beneficial biological properties such as antimicrobial activity (Atkinson & Blakeman, 1982) and the induction of adipogenesis (Saito *et al.*, 2008). *H. taltalense*, is an endemic shrub or bush that grows in arid regions which obtains survival water from condensation from the Chilean coastal fog. Several *Heliotropium* species grow freely mainly in the north of Chile and they have glandular secreting trichomes whose principal rol is to produce a gummy exudate aimed to protect the plant from environmental factors and predators. This exudate is composed mainly of waxes and a mixture of phenolic compounds, mainly flavonoids and aromatic geranyl derivatives (Modak *et al.*, 2009). The title compound (Fig. 1) crystallizes with an organic molecule and two water molecules in the asymmetric unit. The hydroxy group at C5 forms a strong intramolecular hydrogen bond with the carbonyl group with graph-set notation S(6) (Bernstein, *et al.*, 1995). This interaction is observed in the unsolvated compound too, (Shoja, 1990). In the crystal, the components are linked by intermolecular O—H···O hydrogen bonds with an average O···O distance of 2.86 (14) Å and O—H···O angles in the range 147–174°, forming a 3-D network (Fig. 2). The 4'-hydroxyphenyl ring is bonded equatorially to the pyrone ring which adopts a slightly distorted sofa conformation with the C2 atom 0.328 (5) Å out of plane defined by the C2/C3/C4/C9/C10/O1 atoms, [puckering amplitude Q_T_= 0.465 (5) Å; θ =124.9 (6)° & φ = 245.3 (7)° (Cremer & Pople, 1975)]. The title compound is the hydrated form of a previously reported structure. There are only slight variations in the molecular geometry of both compounds, so when both compound are superimposed all atoms are fitted within RMSD 0.0715 Å, (with inversion & flexibility), (Macrae *et al.*, 2008), as is shown in Fig. 3.

### Experimental

Dried aerial parts of *H. taltalense* (1.8 kg) were immersed in ethyl acetate (EtOAc) for one minute (2 l) in order to obtain an extract from the exudate. The extract was immediately concentrated *in vacuo* and the resulting dark brown syrup (47 g) was adsorbed on to silicagel 60 G (50 g) and slurred onto the top of a column containing silica gel 60 H (0.5 kg), partitioned using a medium pressure pump with an isocratic eluent (*n*-hexane–EtOAc 8:2), to obtain six partitions (fractions A to F: *n*-hexane, *n*-hexane–EtOAc 95:5, *n*-hexane–EtOAc 90:10, *n*-hexane–EtOAc 80:20, *n*-hexane–EtOAc 50:50 and pure EtOAc). Further purification by a combination of chromatography on silicagel 60 H and permeation through Sephadex LH-20 (eluting with methanol-water 7:3) of the fraction 20% hexane-ethyl acetate (fraction D, 15 g) afforded the phytoalexin 5,4'-di-hydroxy-7-methoxyflavanone dihydrate for which 1-D and 2-D NMR data are consistent with literature (Agrawal, 1989; Ogawa, *et al.*, 2007). Recrystallization from hexane-ethyl acetate (9:1) at -20 ° C yielded yellow crystals of (*S*)-sakuranetin dihydrate (0.039 g), suitable for X-ray diffraction analysis.

### Refinement

The parameters of the three H atoms bonded to atoms O5 (hydroxyl) and O6 (water) were located in difference maps and refined isotropically; all other H atoms were treated using a riding model, with C—H distances are in the range 0.93 – 0.98 Å and O—H distance of 0.82 and 0.85 Å, with *U*_iso_(H) = 1.2*U*_eq_(C,*O*) or 1.5*U*_eq_(methyl C). The absolute configuration could not be established by the Flack method and the 772 observed Friedel opposites were merged.

### Crystal data

**Table:**

C_16_H_14_O_5_·2H_2_O	*F*(000) = 680
*M**_r_* = 322.30	*D*_x_ = 1.376 Mg m^−^^3^
Orthorhombic, *P*2_1_2_1_2_1_	Mo *K*α radiation, λ = 0.71073 Å
Hall symbol: P 2ac 2ab	Cell parameters from 2419 reflections
*a* = 5.0869 (10) Å	θ = 3.3–28.4°
*b* = 9.4622 (19) Å	µ = 0.11 mm^−^^1^
*c* = 32.318 (7) Å	*T* = 293 K
*V* = 1555.6 (5) Å^3^	Block, yellow
*Z* = 4	0.20 × 0.15 × 0.03 mm

### Data collection

**Table:**

Nonius KappaCCD area-detector diffractometer	1623 reflections with *I* > 2σ(*I*)
Radiation source: fine-focus sealed tube	*R*_int_ = 0.068
graphite	θ_max_ = 28.4°, θ_min_ = 3.3°
φ and ω scans with κ offsets	*h* = 0→6
9743 measured reflections	*k* = 0→12
2021 independent reflections	*l* = −40→42

### Refinement

**Table:**

Refinement on *F*^2^	Primary atom site location: structure-invariant direct methods
Least-squares matrix: full	Secondary atom site location: difference Fourier map
*R*[*F*^2^ > 2σ(*F*^2^)] = 0.075	Hydrogen site location: inferred from neighbouring sites
*wR*(*F*^2^) = 0.180	H atoms treated by a mixture of independent and constrained refinement
*S* = 1.17	*w* = 1/[σ^2^(*F*_o_^2^) + (0.0649*P*)^2^ + 1.4205*P*] where *P* = (*F*_o_^2^ + 2*F*_c_^2^)/3
2021 reflections	(Δ/σ)_max_ < 0.001
224 parameters	Δρ_max_ = 0.38 e Å^−^^3^
0 restraints	Δρ_min_ = −0.29 e Å^−^^3^

### Special details

**Table:**

Geometry. All s.u.'s (except the s.u. in the dihedral angle between two l.s. planes) are estimated using the full covariance matrix. The cell s.u.'s are taken into account individually in the estimation of s.u.'s in distances, angles and torsion angles; correlations between s.u.'s in cell parameters are only used when they are defined by crystal symmetry. An approximate (isotropic) treatment of cell s.u.'s is used for estimating s.u.'s involving l.s. planes.
Refinement. Refinement of *F*^2^ against ALL reflections. The weighted *R*-factor *wR* and goodness of fit *S* are based on *F*^2^, conventional *R*-factors *R* are based on *F*, with *F* set to zero for negative *F*^2^. The threshold expression of *F*^2^ > 2σ(*F*^2^) is used only for calculating *R*-factors(gt) *etc*. and is not relevant to the choice of reflections for refinement. *R*-factors based on *F*^2^ are statistically about twice as large as those based on *F*, and *R*- factors based on ALL data will be even larger.

### Fractional atomic coordinates and isotropic or equivalent isotropic displacement parameters (Å^2^)

**Table:**

	*x*	*y*	*z*	*U*_iso_*/*U*_eq_	
O1	0.1698 (7)	0.3272 (3)	0.39766 (8)	0.0317 (8)	
O2	−0.3650 (7)	0.5120 (4)	0.31855 (10)	0.0370 (9)	
O3	−0.1585 (7)	0.3656 (4)	0.25747 (9)	0.0350 (8)	
H3	−0.2576	0.4224	0.2686	0.053*	
O4	0.5380 (7)	0.0576 (4)	0.28692 (9)	0.0341 (8)	
O5	0.1305 (11)	0.5341 (6)	0.58135 (12)	0.0610 (15)	
H5	0.247 (18)	0.564 (8)	0.593 (2)	0.07 (3)*	
C2	0.0708 (9)	0.4690 (5)	0.40716 (13)	0.0266 (10)	
H2	0.1893	0.5381	0.3945	0.032*	
C3	−0.2023 (9)	0.4877 (6)	0.38806 (13)	0.0345 (12)	
H3A	−0.3254	0.4250	0.4018	0.041*	
H3B	−0.2613	0.5840	0.3924	0.041*	
C4	−0.2002 (9)	0.4562 (5)	0.34223 (13)	0.0265 (10)	
C10	−0.0071 (9)	0.3561 (5)	0.32828 (12)	0.0247 (9)	
C5	0.0125 (9)	0.3128 (5)	0.28607 (12)	0.0248 (10)	
C6	0.1952 (10)	0.2153 (5)	0.27344 (13)	0.0278 (10)	
H6	0.2063	0.1899	0.2457	0.033*	
C7	0.3658 (9)	0.1537 (5)	0.30263 (13)	0.0251 (9)	
C8	0.3505 (9)	0.1902 (5)	0.34455 (13)	0.0267 (9)	
H8	0.4586	0.1465	0.3639	0.032*	
C9	0.1720 (8)	0.2925 (5)	0.35667 (12)	0.0221 (9)	
C1'	0.0839 (9)	0.4870 (5)	0.45324 (13)	0.0265 (10)	
C2'	−0.0739 (11)	0.4059 (6)	0.48002 (14)	0.0379 (12)	
H2'	−0.1923	0.3410	0.4691	0.046*	
C3'	−0.0542 (12)	0.4220 (6)	0.52256 (15)	0.0439 (14)	
H3'	−0.1581	0.3673	0.5400	0.053*	
C4'	0.1203 (10)	0.5197 (6)	0.53925 (13)	0.0367 (12)	
C5'	0.2769 (12)	0.6004 (6)	0.51347 (15)	0.0441 (14)	
H5'	0.3940	0.6656	0.5246	0.053*	
C6'	0.2585 (11)	0.5837 (6)	0.47061 (14)	0.0361 (12)	
H6'	0.3645	0.6381	0.4534	0.043*	
C11	0.7076 (11)	−0.0153 (6)	0.31527 (15)	0.0364 (12)	
H11A	0.6039	−0.0734	0.3334	0.055*	
H11B	0.8043	0.0523	0.3314	0.055*	
H11C	0.8280	−0.0737	0.3001	0.055*	
O6	0.2193 (9)	0.7134 (5)	0.32036 (13)	0.0500 (11)	
H6A	0.354 (18)	0.659 (8)	0.316 (2)	0.07 (2)*	
H6B	0.209 (15)	0.761 (6)	0.2996 (19)	0.06 (2)*	
O7	0.5931 (9)	0.6486 (6)	0.60698 (12)	0.0586 (13)	
H7A	0.7450	0.6441	0.5961	0.088*	
H7B	0.6306	0.6736	0.6315	0.088*	

### Atomic displacement parameters (Å^2^)

**Table:**

	*U*^11^	*U*^22^	*U*^33^	*U*^12^	*U*^13^	*U*^23^
O1	0.0332 (19)	0.0370 (19)	0.0248 (15)	0.0105 (18)	−0.0035 (13)	−0.0066 (13)
O2	0.0244 (17)	0.046 (2)	0.0406 (18)	0.0133 (18)	−0.0064 (14)	0.0006 (16)
O3	0.0334 (19)	0.039 (2)	0.0324 (16)	0.0081 (19)	−0.0069 (14)	0.0014 (14)
O4	0.0292 (18)	0.041 (2)	0.0324 (16)	0.0126 (17)	−0.0009 (14)	−0.0085 (14)
O5	0.061 (3)	0.096 (4)	0.0258 (18)	−0.028 (3)	0.0028 (19)	−0.006 (2)
C2	0.018 (2)	0.032 (2)	0.030 (2)	0.001 (2)	0.0035 (17)	−0.0057 (18)
C3	0.022 (2)	0.044 (3)	0.038 (2)	0.012 (3)	0.0049 (19)	−0.006 (2)
C4	0.018 (2)	0.029 (2)	0.032 (2)	0.000 (2)	−0.0033 (18)	0.0001 (18)
C10	0.018 (2)	0.028 (2)	0.028 (2)	−0.001 (2)	0.0005 (17)	−0.0006 (17)
C5	0.023 (2)	0.027 (2)	0.025 (2)	−0.005 (2)	−0.0066 (17)	0.0041 (17)
C6	0.032 (2)	0.031 (2)	0.0204 (19)	0.002 (2)	0.0008 (18)	−0.0029 (17)
C7	0.017 (2)	0.026 (2)	0.032 (2)	0.002 (2)	0.0021 (17)	−0.0008 (18)
C8	0.023 (2)	0.031 (2)	0.026 (2)	0.002 (2)	−0.0012 (17)	0.0009 (18)
C9	0.0115 (18)	0.029 (2)	0.0259 (19)	−0.0042 (19)	−0.0017 (15)	−0.0002 (17)
C1'	0.022 (2)	0.029 (2)	0.028 (2)	−0.001 (2)	−0.0004 (17)	−0.0030 (18)
C2'	0.032 (3)	0.048 (3)	0.033 (2)	−0.008 (3)	−0.002 (2)	−0.006 (2)
C3'	0.039 (3)	0.054 (4)	0.039 (3)	−0.012 (3)	0.012 (2)	0.003 (2)
C4'	0.034 (3)	0.050 (3)	0.027 (2)	−0.001 (3)	0.005 (2)	0.000 (2)
C5'	0.039 (3)	0.057 (4)	0.036 (3)	−0.008 (3)	−0.003 (2)	−0.009 (2)
C6'	0.029 (3)	0.047 (3)	0.031 (2)	−0.012 (2)	0.0050 (19)	−0.001 (2)
C11	0.027 (3)	0.040 (3)	0.042 (3)	0.014 (3)	0.000 (2)	−0.001 (2)
O6	0.048 (3)	0.058 (3)	0.044 (2)	0.020 (2)	0.007 (2)	0.001 (2)
O7	0.049 (3)	0.084 (3)	0.042 (2)	−0.008 (3)	−0.0053 (19)	−0.007 (2)

### Geometric parameters (Å, °)

**Table:**

O1—C9	1.365 (5)	C7—C8	1.400 (6)
O1—C2	1.465 (5)	C8—C9	1.383 (6)
O2—C4	1.252 (5)	C8—H8	0.9300
O3—C5	1.364 (5)	C1'—C6'	1.393 (6)
O3—H3	0.8200	C1'—C2'	1.408 (7)
O4—C7	1.361 (5)	C2'—C3'	1.387 (7)
O4—C11	1.435 (6)	C2'—H2'	0.9300
O5—C4'	1.369 (6)	C3'—C4'	1.391 (7)
O5—H5	0.75 (8)	C3'—H3'	0.9300
C2—C1'	1.501 (6)	C4'—C5'	1.382 (7)
C2—C3	1.530 (6)	C5'—C6'	1.397 (6)
C2—H2	0.9800	C5'—H5'	0.9300
C3—C4	1.511 (6)	C6'—H6'	0.9300
C3—H3A	0.9700	C11—H11A	0.9600
C3—H3B	0.9700	C11—H11B	0.9600
C4—C10	1.437 (6)	C11—H11C	0.9600
C10—C9	1.426 (6)	O6—H6A	0.87 (9)
C10—C5	1.427 (6)	O6—H6B	0.81 (6)
C5—C6	1.372 (6)	O7—H7A	0.8500
C6—C7	1.408 (6)	O7—H7B	0.8501
C6—H6	0.9300		
			
C9—O1—C2	115.3 (3)	C9—C8—H8	120.6
C5—O3—H3	109.5	C7—C8—H8	120.6
C7—O4—C11	118.0 (4)	O1—C9—C8	116.7 (4)
C4'—O5—H5	123 (6)	O1—C9—C10	121.2 (4)
O1—C2—C1'	107.3 (4)	C8—C9—C10	122.2 (4)
O1—C2—C3	109.5 (4)	C6'—C1'—C2'	118.3 (4)
C1'—C2—C3	115.3 (4)	C6'—C1'—C2	120.2 (4)
O1—C2—H2	108.2	C2'—C1'—C2	121.5 (4)
C1'—C2—H2	108.2	C3'—C2'—C1'	120.6 (5)
C3—C2—H2	108.2	C3'—C2'—H2'	119.7
C4—C3—C2	111.5 (4)	C1'—C2'—H2'	119.7
C4—C3—H3A	109.3	C2'—C3'—C4'	120.2 (5)
C2—C3—H3A	109.3	C2'—C3'—H3'	119.9
C4—C3—H3B	109.3	C4'—C3'—H3'	119.9
C2—C3—H3B	109.3	O5—C4'—C5'	121.5 (5)
H3A—C3—H3B	108.0	O5—C4'—C3'	118.4 (5)
O2—C4—C10	123.0 (4)	C5'—C4'—C3'	120.1 (5)
O2—C4—C3	120.7 (4)	C4'—C5'—C6'	119.8 (5)
C10—C4—C3	116.3 (4)	C4'—C5'—H5'	120.1
C9—C10—C5	116.7 (4)	C6'—C5'—H5'	120.1
C9—C10—C4	120.9 (4)	C1'—C6'—C5'	121.1 (4)
C5—C10—C4	122.4 (4)	C1'—C6'—H6'	119.5
O3—C5—C6	118.5 (4)	C5'—C6'—H6'	119.5
O3—C5—C10	119.9 (4)	O4—C11—H11A	109.5
C6—C5—C10	121.6 (4)	O4—C11—H11B	109.5
C5—C6—C7	119.8 (4)	H11A—C11—H11B	109.5
C5—C6—H6	120.1	O4—C11—H11C	109.5
C7—C6—H6	120.1	H11A—C11—H11C	109.5
O4—C7—C8	124.2 (4)	H11B—C11—H11C	109.5
O4—C7—C6	115.0 (4)	H6A—O6—H6B	105 (6)
C8—C7—C6	120.8 (4)	H7A—O7—H7B	101.2
C9—C8—C7	118.9 (4)		
			
C9—O1—C2—C1'	−180.0 (4)	C2—O1—C9—C8	155.2 (4)
C9—O1—C2—C3	54.2 (5)	C2—O1—C9—C10	−26.6 (6)
O1—C2—C3—C4	−54.4 (5)	C7—C8—C9—O1	−178.2 (4)
C1'—C2—C3—C4	−175.4 (4)	C7—C8—C9—C10	3.6 (6)
C2—C3—C4—O2	−153.5 (5)	C5—C10—C9—O1	179.7 (4)
C2—C3—C4—C10	28.6 (6)	C4—C10—C9—O1	−1.7 (6)
O2—C4—C10—C9	−178.3 (4)	C5—C10—C9—C8	−2.2 (6)
C3—C4—C10—C9	−0.5 (6)	C4—C10—C9—C8	176.4 (4)
O2—C4—C10—C5	0.3 (7)	O1—C2—C1'—C6'	112.6 (5)
C3—C4—C10—C5	178.0 (4)	C3—C2—C1'—C6'	−125.2 (5)
C9—C10—C5—O3	177.7 (4)	O1—C2—C1'—C2'	−66.1 (6)
C4—C10—C5—O3	−0.9 (7)	C3—C2—C1'—C2'	56.1 (6)
C9—C10—C5—C6	−0.2 (6)	C6'—C1'—C2'—C3'	−0.3 (8)
C4—C10—C5—C6	−178.8 (4)	C2—C1'—C2'—C3'	178.5 (5)
O3—C5—C6—C7	−176.9 (4)	C1'—C2'—C3'—C4'	0.6 (8)
C10—C5—C6—C7	1.1 (7)	C2'—C3'—C4'—O5	178.9 (5)
C11—O4—C7—C8	2.6 (7)	C2'—C3'—C4'—C5'	−0.6 (9)
C11—O4—C7—C6	−176.5 (4)	O5—C4'—C5'—C6'	−179.3 (5)
C5—C6—C7—O4	179.5 (4)	C3'—C4'—C5'—C6'	0.2 (9)
C5—C6—C7—C8	0.4 (7)	C2'—C1'—C6'—C5'	−0.1 (8)
O4—C7—C8—C9	178.2 (4)	C2—C1'—C6'—C5'	−178.9 (5)
C6—C7—C8—C9	−2.7 (7)	C4'—C5'—C6'—C1'	0.2 (9)

### Hydrogen-bond geometry (Å, °)

**Table:**

*D*—H···*A*	*D*—H	H···*A*	*D*···*A*	*D*—H···*A*
O3—H3···O2	0.82	1.90	2.630 (5)	147
O5—H5···O7	0.76 (9)	1.99 (9)	2.720 (7)	163 (7)
O6—H6A···O2^i^	0.87 (9)	2.00 (8)	2.847 (6)	166 (7)
O6—H6B···O3^ii^	0.81 (6)	2.11 (6)	2.915 (5)	174 (8)
O7—H7A···O5^i^	0.85	2.27	3.055 (7)	153
O7—H7B···O6^iii^	0.85	1.94	2.763 (6)	163

Symmetry codes: (i) *x*+1, *y*, *z*; (ii) −*x*, *y*+1/2, −*z*+1/2; (iii) *x*+1/2, −*y*+3/2, −*z*+1.
